# Is there a role of faecal microbiota transplantation in reducing antibiotic resistance burden in gut? A systematic review and Meta-analysis

**DOI:** 10.1080/07853890.2021.1927170

**Published:** 2021-06-25

**Authors:** Priyanga Dharmaratne, Nannur Rahman, Anthony Leung, Margaret Ip

**Affiliations:** a Faculty of Medicine, Department of Microbiology, The Chinese University of Hong Kong, Prince of Wales Hospital, Sha Tin, China; b Shenzhen Research Institute, The Chinese University of Hong Kong, Shenzhen, China

**Keywords:** Faecal microbiota transplantation, antibiotic resistance, systematic review and meta-analysis, case study, case series study

## Abstract

**Objectives:**

The aim of current systematic review and meta-analysis is to provide insight into the therapeutic efficacy of fecal microbiota transplantation (FMT) for the decolonization of antimicrobial-resistant (AMR) bacteria from the gut.

**Methods:**

The protocol for this Systematic Review was prospectively registered with PROSPERO (CRD42020203634). Four databases (EMBASE, MEDLINE, SCOPUS, and WEB of SCIENCE) were consulted up until September 2020. A total of fourteen studies [*in vivo* (*n* = 2), case reports (*n* = 7), case series without control arm (*n* = 3), randomized clinical trials (RCT, *n* = 2)], were reviewed. Data were synthesized narratively for the case reports, along with a proportion meta-analysis for the case series studies (*n* = 102 subjects) without a control arm followed by another meta-analysis for case series studies with a defined control arm (*n* = 111 subjects) for their primary outcomes.

**Results:**

Overall, seven non-duplicate case reports (*n* = 9 participants) were narratively reviewed and found to have broad AMR remission events at the 1-month time point. Proportion meta-analysis of case series studies showed an overall 0.58 (95% CI: 0.42-0.74) AMR remission. Additionally, a significant difference in AMR remission was observed in FMT vs treatment naïve (RR = 0.44; 95% CI: 0.20-0.99) and moderate heterogeneity (*I*^2^=65%). A subgroup analysis of RCTs (*n* = 2) revealed FMT with further benefits of AMR remission with low statistical heterogeneity (RR = 0.37; 95% CI: 0.18-0.79; *I*^2^ =23%).

**Conclusion:**

More rigorous RCTs with larger sample size and standardized protocols on FMTs for gut decolonization of AMR organisms are warranted.

**KEY MESSAGE**
- Existing studies in this subject are limited and of low quality with moderate heterogeneity, and do not allow definitive conclusions to be drawn.
- More rigorous RCTs with larger sample size and standardized protocols on FMTs for gut decolonization of AMR organisms are warranted.

## Introduction

Currently, antimicrobial resistance (AMR) has been identified as one of the major threats to global health, food production, and economic development [1]. The US Centres for Disease Control and Prevention has estimated that each year, >2.8 M patients are infected with antibiotic-resistant (AR) bacteria and >35,000 dies of these infections. Also, nearly 223,900 people in the United States required hospital care for *C. difficile* and at least 12,800 people died in 2017 [2]. AMR is most often conferred through the expression of antimicrobial resistance genes that reduce a microbe’s susceptibility to the effects of antibiotics. AMR bacteria are stratified to, *Enterococcus faecium*, *Staphylococcus aureus*, *Klebsiella pneumoniae*, *Acinetobacter baumannii*, *Pseudomonas aeruginosa*, and *Enterobacter* species, known to be “ESKAPE”, are responsible for the majority of hospital infections with higher mortality rates [3]. Data on AMR in Europe are reported by the European Antimicrobial Resistance Surveillance Network (EARS-Net) 2018 report, stated that more than half (58.3%) of the *E. coli* and third (37.2%) of *K. pneumoniae* isolates responsible for invasive diseases were resistant to at least one of the antimicrobial groups under regular surveillance (i.e. aminoglycosides, aminopenicillins, fluoroquinolones, third-generation cephalosporins, and carbapenems) [4].

The human gastrointestinal (GI) tract is colonized by different kinds of bacteria, archaea, fungus, and viruses, consensually termed as gut microbiota [5]. The intestinal microbiome of healthy patients often consists of well-balanced diversified microbiota members that predominantly belong to just four phyla—the Bacteroidetes, Firmicutes, Actinobacteria, and Proteobacteria and known to pose colonization resistance (CR) [6]. Gut microbiota can produce a variety of compounds that play key roles in the colon micro-ecology and host homeostasis. Delicate contemporary approaches to unravel the importance of the symbiosis of gut microbiota lead to its identification as a potential target of many chronic diseases ranging from gastrointestinal inflammatory and metabolic conditions to neurological, respiratory, and cardiovascular illnesses [7]. Apart from that, gut microbiota plays a beneficial role in maintaining human health via producing short-chain fatty acids (SCFA), vitamins and acting as a shield to protect the host from colonization by pathogenic bacteria [8].

**Figure 1.**
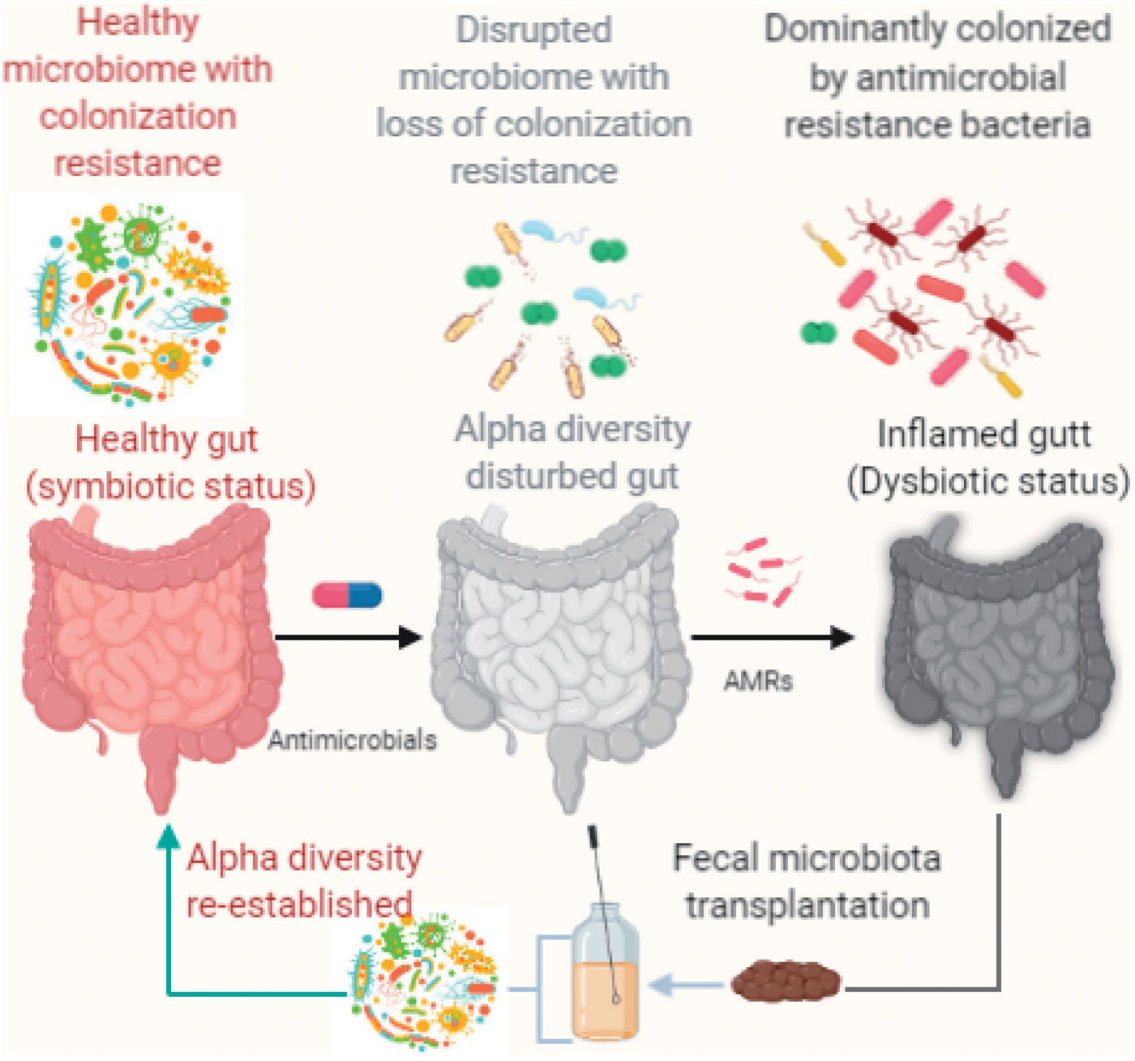
Concept illustration of colonization resistance due to alpha diversity of gut microbes and the effect of antimicrobials in destabilization of symbiotic stage. The disturbed gut microbiota could be colonized with AMRs and leads to dysbiosis. FMT is an alternative therapeutic modality to restore the alpha diversity by decolonizing AMRs.

However, under certain circumstances, the patient may develop a compromised microbiota by declining alpha-composition and it causes CR vulnerability and eventually leads to exogenous bacterial colonization. The deleterious effects of antibiotics on gut microbiota have been extensively studied [9,10]. Other than antibiotics, proton pump inhibitors (PPIs) are one of the most commonly prescribed drugs in western medicine [11], and they lead to a profound and prolonged reduction of gastric acid production. The association of PPIs and the risk of some enteric infections namely, *Clostridium difficile*, *Campylobacter*, *Salmonella* are well documented [12–14]. Importantly, antipsychotic drugs such as olanzapine [15], pimozide [16], fluphenazine [17], and flupenthixol dihydrochloride [18] were proven to pose *in vitro* antibacterial properties against a broad spectrum of bacteria alone and in combination with other antibiotics. A significant population of the community consumes these drugs and they may have disturbed gut microbiota composition, and consequently put their CR at risk. Hence, their intestinal microbiota dysbiosis provides favourable conditions for AMR bacteria to colonize and eventually act as a reservoir for horizontal resistant gene transfer [19,20]. Horizontal gene transfer has been documented as an important mechanism for the transfer and acquisition of antimicrobial resistance genes within and between gut bacterial species (Figure 1) [21–23].

Research on the bilateral relationship between gut microbiota and human health has been in the spotlight during the last decade. This has been mainly driven forward by improved next-generation sequencing (NGS) technologies [24] and novel proteomic [25] approaches, allowing the profiling of entire microbial communities with high efficacy and low cost.

The conservation effect of gut microbiota from AR has encouraged scientists to study faecal microbiota transplantation (FMT) to restore a healthy gut microbiome by eliminating AR colonization. The concept behind FMT is to directly alter the gut microbial composition of the recipient to establish the alpha-diversity [26]. This is achieved via the administration of a frozen, encapsulated or fresh solution of faecal matter from a donor into the intestinal tract of a recipient to confer a health benefit via altering the gut microbial composition [27]. The initial step of the process involves a thorough screening procedure to identify a suitable donor. This includes a questionnaire of the donors’ family health history, contemporary exposure to any medication and a series of laboratory tests to ensure there is no transmittable disease or pathogens [28]. According to the contemporary guidelines from the Infectious Diseases Society of America (IDSA) and the Society of Healthcare Epidemiology of America (SHEA), as well as the European Consensus Guidelines, FMT is recommended as a second-line treatment modality against recurrent *C. difficile* infection, due to over 90% efficacy in randomized control trials [26]. But the application of FMT for the decolonization of AMR microorganisms other than *C. difficile* is controverisal due to a limited number of reports [29–36].

The main objective of our review is to descriptively analyze the non-duplicate data and pool all published data to ascertain a conclusive statistical picture of the primary outcome, set as the decolonization of AMRs (except recurrent *C. difficile*) in adults (>18 years old) by FMT intervention at the 1-month time point. Secondary outcomes are to provide systematic descriptive analyses of case studies on decolonization of AMR via FMT, identifying adverse effects associated with FMT procedures, and outline key steps to be followed in future rigorous clinical trials.

## Methods

### Protocol development

We registered our review protocol (CRD42020203634) in the International Prospective Register of Systematic Reviews (PROSPERO) that is available at https://www.crd.york.ac.uk/prospero/display_record.php?ID=CRD42020203634. We adhered to the recommendations of the PRISMA-P 2015 statement in developing this protocol and conducting the review [37].

### Search strategy

This global search was performed using four bibliographic databases, namely, EMBASE and MEDLINE through PubMed, SCOPUS, and WEB of SCIENCE. The following search terms were used: (“Faecal microbiota transplantation” OR “FMT” OR “bacteriotherapy” OR “gut microbiota transplantation” OR “fecal transplantation” OR “intestinal microbiota transfer”) AND (“intestinal antibiotic-resistant bacteria” OR “antibiotic-resistant bacteria” OR “intestinal antimicrobial resistance” OR “AMR”). The same search term was used in all databases to retrieve the data for this review. Apart from that, we have searched the following clinical trial registries; United States (www.clinicaltrials.org), Australia-New Zealand (www.anzctr.org.au) United Kingdom (www.isrctn.com), Germany (www.drks.de), and China (www.chictr.org.cn)

The final database update was carried out on 15 September 2020.

### Eligibility criteria

#### Types of studies

This review included studies that investigated the effectiveness of FMT in eliminating AMR colonization confined to the gut. As a fact, FMT is an emerging therapy for the decolonization of AMR for intestinal carriage, hence the data on randomized clinical trials are scanty. Thus, we included different types of clinical trials including, cohort studies, case studies, case series studies, and case reports. Apart from clinical investigations, we also reviewed *in vivo* studies dealing with the same purpose. We excluded all the review articles, news, conference proceedings, editorials, and letters to the editor, expert opinions, or commentaries as they did not provide adequate information for review. Except for case series studies (used only for the meta-analysis), we also excluded studies (case reports) that were previously reviewed.

#### Types of participants and eligible AMR bacteria

The adult population (>18 years) was included in the review. Studies with paediatric patients were excluded since their gut microbiome is in a dynamically developing stage and the study outcome could not be compared with the adults [38], and those who are carrying rather stable gut microbiome [39]. Patients who received antibiotics concurrently at the time the FMT were also excluded, due to its direct effect on the composition of the transplanting bacteria [40]. However, patients whose concomitant antibiotic therapy was discontinued at least 24 h before the FMT procedure were included.

AMRs involving viruses and fungi and AMR bacteria colonized outside the gut were also excluded. Furthermore, FMT on the patients who had recurrent or refractory *C. difficile* infections alone was also excluded from our review process, since there are other reviews solely focussed on the topic [41–43].

#### Types of interventions and outcome measures

The single-arm intervention trials were abundant in numbers in this review, but studies with control groups including placebo, antibiotics, or treatment-naive were also included, where necessary. Different FMT administration routes (upper and lower gastrointestinal routes) were considered including; caecum through colonoscopy, oral gavage, naso-duodenal tube and delivered via enema. The fresh, frozen or encapsulated samples were used in FMT along with related or unrelated donors, as well as, single or multiple FMTs were included in the review.

The primary outcome measured was the decolonization of AMR within 1 month (30 days) upon FMT with two consecutive negative results of rectum swabs or confirmed by PCR. However, the inter-study variation could be observed in the outcome for decolonization testing and are included in this review.

### Assessment of risk of bias of eligibility criteria for the articles

This assessment was performed in several ways including, the use of https://www.covidence.org online platform. At the beginning, all the citations (from 4 databases) were uploaded to the software in “RIS” file format. A preliminary abstract screening was carried out by NR and AL for inclusion and the selected studies were subjected to scrutiny by full-text review by NR, AL, and PD to avoid the risk of bias. Any disparities were resolved upon the consultation of senior author MI.

Moreover, the selected studies were further verified by NR and PD, according to the Joanna Briggs Institution (JBI) standardized critical appraisal checklists for case series and case reports for the possibility of bias in its design, conduct, and analysis [44,45]. The completed JBI forms for the included studies were given in Supplementary information, Section 1. The studies with selection “No” for at least 3 questions in the appraisal form considered to pose a lack of integrity in the study design, hence excluded from data abstraction and reviewing.

Overall completeness and transparency of the included case reports were verified using CARE guidelines [46] and completed forms for the included studies are given in Supplementary information, Section 2. Similarly, PROCESS guidelines [47] were used to verify the quality and risk of bias of the case series studies (Supplementary information, Section 3). Quality of evidence for two randomized control trials and the remaining 3 case series studies were assessed following the GRADE recommendations [48] and completed on the GRADEpro online software (Supplementary information, Section 4).

### Data abstraction

We developed a data abstraction spreadsheet using Excel version 2016 software (Microsoft Corporation, Redmond, Washington, USA). We conducted the data abstraction for the included full-text articles, and the data were independently assessed by two review authors. We extracted the following information: title, DOI number, type of study, acceptance or rejection of topic, type of faecal material, methods of infusion, characteristics or composition of stool, types of patients, the role of FMT on the antibiotic-resistant bacteria, mode of action, and side-effects with potential remarks.

## Data synthesis

Two separate meta-analyses were carried out for the case series studies with and without control arms for their primary objective (percentage decolonization at the 1-month time point). However, in two studies [49,50], the decolonization rate was considered at the 35 to 48-day time point. The case reports were excluded from the meta-analysis, since the single data is not adequate to provide the estimation of effect size and for which, we have provided only a narrative synthesis [51].

The outcome for the case series studies is dichotomous with only two responses, such as AMR decolonization was successful or not. The meta-analysis for the case series studies without a control arm was analysed as proportions of decolonization [52,53] under the random-effects model using StatsDirect v3 statistical software. Analyses of the case series studies with the inclusion of a proper treatment naïve group were conducted via RevMan 5.4 using a random-effects model. For both meta-analyses, the combined effect was illustrated via creating a forest plot.

A statistically significant *p*-value was based on *p* < .05. Clinical and methodological heterogeneity across the included studies were expressed descriptively, while the statistical heterogeneity between the studies included for the two meta-analyses was expressed by using the *I*^2^ statistic, while *I*^2^ values interpreted as; low, moderate, and high levels of heterogeneity where, *I*^2^ < 50%, *I*^2^ 50–75%, and *I*^2^ > 75%, respectively [54].

## Results

### Study selection

**Figure 2.**
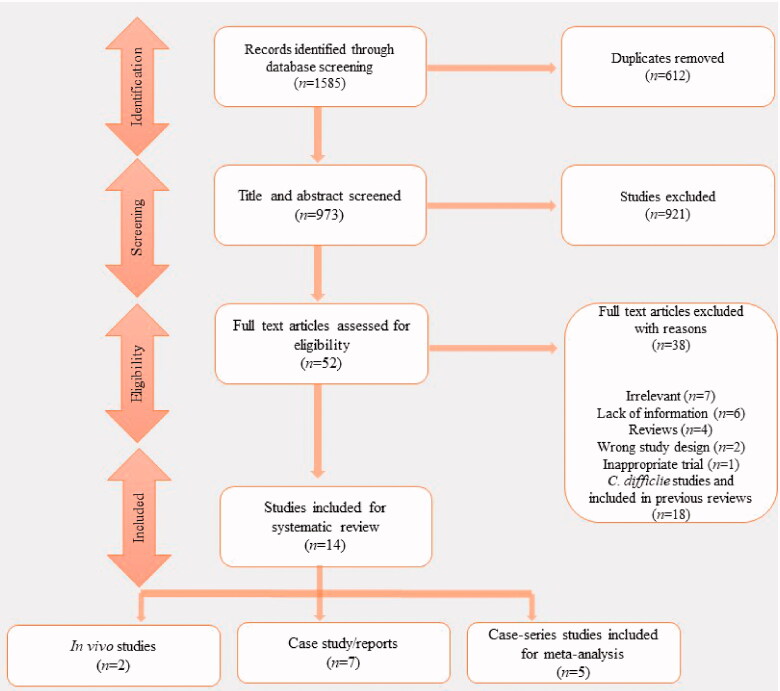
Flowchart showing the systematic review process with the bibliometric assessment, including article attrition and study selection. Briefly, articles were filtered through an automated bibliographic database search using keywords.

The search strategy from EMBASE and MEDLINE via PubMed, SCOPUS, and WEB of SCIENCE yielded one thousand five hundred and eighty-five (*n* = 1585) studies, none of the studies were retrieved from the grey literature. Upon removal of duplicates (*n* = 612), the title and abstracts of nine hundred and seventy-three (*n* = 973) studies were screened, and nine hundred and twenty-one (*n* = 921) studies did not meet the selection criteria. The full texts of the remaining fifty-two (*n* = 52) studies were assessed against the pre-defined inclusion and exclusion criteria, but thirty-eight (*n* = 38) studies did not meet the ultimate inclusion criteria. Additionally, the studies already included in previous literature reviews [38–40,50] were also excluded in abstracting data or review in detail. However, previously published case series studies were included in 2 meta-analyses to get an overall statistical conclusion on the effect of FMT on the decolonization of AMRs at the 1-month time point. Finally, fourteen (*n* = 14) studies were eligible for this detailed data abstraction and the broad discussion. The process of the selection of studies is summarized in a PRISMA flow diagram (Figure 2). Three reviewers, NR, AL, and PD, screened the studies for inclusion and exclusion in the systematic review and meta-analysis and all the review authors mutually agreed on the final set of articles to be included.

### Characteristics of included studies

The eligible articles had a publication date from September 2015 to September 2020. Out of fourteen studies, the majority are case reports or case studies (*n* = 7) followed by different types of case series (*n* = 5) and two (*n* = 2) *in vivo* studies involving mice models. Out of the five case series studies, two (*n* = 2) were carried out in multiple centres (Switzerland, Netherland, France, and Israel) [49,50], while the remaining (*n* = 3) are single-center studies [55–57]. Most of the case reports/studies (*n* = 5/7) were carried out in Europe; namely, Denmark (*n* = 2) [58,59], Poland (*n* = 2) [60,61], Netherland (*n* = 1) [62], and one study each in USA [63] and South Africa [64]. AMR colonization in the gut was varied among studies, particularly in CREs. Except in one case study where the gut was colonized by MDR *Salmonella infantis*, multiple MDR bacteria colonized participants across all other studies [63]. The clinical diagnostics of the subjects who underwent FMT across the case reports/studies included patients with; immunocompromised [61], diabetic [62], renal transplant [58], different types of leukemia [60,63] and critically ill with multiple organ failure [59,64]. We have observed an inter-study variability in using PPIs. None of the case studies/reports used PPIs in the pre-treatment step, except in Bilinki et al. [61]. This observation was in contrast to that in case-series studies, where PPIs (omeprazole and pentaprazole) were used in all studies as a pre-treatment in different time points, except in Leo et al. [49] Bowel cleansing was not conducted in any of the case studies/reports, but it has been applied in many case series studies [55–57]. Except in one case study [64], the FMTs in all included studies were procured from unrelated healthy donors. Among most case studies/reports (*n* = 5/7), fresh stool samples were used and diluted with sodium chloride solutions (saline). In contrast, FMT was applied as capsules from frozen stool samples (*n* = 3/5) with a dosage of 15 capsules/day for two consecutive days, in case series studies [49,50,55]. Among capsule-based FMTs, one study used 80% of glycerol instead of 10% in preparation of the capsule, to increase the stability [50].

### Application of FMT in AMR decolonization in gut

#### *In vivo* models

The study characteristics of the two eligible *in vivo* studies are given in Table 1. FMT is effective in decolonizing various common resistant pathogens including, MDR *P. aeruginosa*, and concurrent colonization of vancomycin-resistant *E. faecium* and KPC [65,66]. Both included studies have shown that FMT significantly decreased the faecal load (in terms of CFU) of colonizing resistant bacteria when compared to those that did not receive FMT [65,66].

**Table 1.** Characteristics of the *in vivo* studies eligible for the review.

	Mrazek et al. [65]	Caballero et al. [66]
In *vivo* model	8-weeks old female C57BL/6j mice	6–8-week-old C57BL/6 female mice
MDR bacteria, resistant gene/pattern, load injected	MDR *P. aeruginosa*, sensitive to fosfomycin and colistin only, 10^9^ CFU on 2 consecutive days	*E. faecium* *K. pneumoniae*, Vancomycin and carbapenem resistant, 5 × 10^4^ CFU by oral gavage
Confirmation of infection	Stool culture for CFU and 16 s rRNA analysis	Stool culture for CFU, 16 s rRNA analysis, Fluorescence *in-situ* hybridisation
FMT donor	5 healthy human or 10 age and sex matched specific pathogen-free control mice	Untreated mice
FMT sample preparation	Dissolved in sterile PBS, aliquoted, and stored a*t* − 80 °C. Immediately before FMT, individual faecal aliquots were thawed and pooled.	Faecal pellet/1 ml of PBS
FMT route and FMT dose	Oral gavage, 0.3 ml for 3 consecutive days	Oral gavage, 200 μl portion three doses
FMT efficacy	4 log reduction of bacteria was observed at 1 W time point, irrespective of source of the donor	*K. pneumoniae* density in faecal pellets decreased within one day and became undetectable within 7 days in all mice

A 2.5 and 4 log_10_ average CFU reduction was observed in MDR *P. aeruginosa* level in mice receiving FMT by murine donors and human donors, respectively, within 7 days post-FMT [65], implying donors of the same species can produce a stronger colonization resistance [65].

KPC was cleared more effectively than VRE in mice receiving FMT (100% and 60% clearance for *K. pneumoniae* and VRE, respectively) [66]. It may suggest there are different mechanisms of colonization resistance against VRE and *K. pneumoniae*, or *K. pneumoniae* is more susceptible to colonization resistance [66].

Except for the FMT donor, the mice model, colonization confirmation, and the route of FMT administration were quite similar in both studies (Table 1). However, the dosage of FMT and target MDR bacterial species vary among studies.

#### Case reports of FMT

The study characteristics of the seven case studies/reports included for the review are given in Table 2. In the case reports, the patients were mostly colonized with Enterobacteriaceae (Table 2). Besides, some other MDR pathogens were also confirmed such as; *Candida albicans, Candida parapsilosis, Enterococcus faecalis, E. faecium,* and *S. infantis* [58,60,62–64]. Except Ueckermann et al. [64], for the reaming studies, the colonization confirmation was achieved via rectal swab analysis

**Table 2.** Characteristics of published case study/reports describing outcomes of FMT against AMR Decolonization.

		Biliński et al. [61]	Stalenhoef et al. [62]	Grosen et al. [58]	Soto et al. [64]	Biernat et al. [60]	Bahl et al. [59]	Ueckermann et al. [64]
Type of study		Case study	Case report	Case report	Case study	Case report	Case study	Case report
	Status	Immuno- compromised patient	Diabetic patient with recurrent UTIs	Recurrent UTI in first five months after Renal transplant recipient (RTX)	Patient 1 (P1): chronic lymphocytic leukaemia Patient 2 (P2); chronic arthropathy on rituximab	P1: acute myeloid leukaemia P2: osteomyelofibrosis	Multi organ failure along with severe recurrent CDI	Critically ill patient
	Age	51 years	34 years	64 years	P1: 60 years P2: 48 years	P1: 25 years P2: 32 years	69 years	60 years
	M/F	M	M	M	P1: M P2: F	P1: M P2: M	F	M
Indication/colonization status and virulence genes		*K. pneumoniae* NDM^+^ and *E. coli* ESBL	MDR *P.aeruginosa* and MDR *E. coli*	ESBL+ *K. pneumoniae*	P1: MDR *S. infantis* P1: MDR *S. infantis*	P1: *E. faecalis, C. albicans and ESBL + E. coli and K. pneumoniae P2: ESBL+ E. coli E. faecium GRE, C. albicans, C. parapsilosis, K. pneumonia etc.*	*C. difficilli* and KPC-producing, XDR *K. pneumoniae*	MDR *K. pneumoniae* Infection+ *Candida parapsilosis*
Colonization confirmation		Rectal swab	Rectal swab	Stool sample	Stool sample	Stool sample	Stool sample	Blood cultures
		Biliński et al. [5,61]	Stalenhoef et al. [62]	Grosen et al. [58]	Soto et al. [63]	Biernat et al. [60]	Bahl et al. [59]	Ueckermann et al. [64]
Pre treatment	Antibiotic	penicillin V, co-trimoxazole	colistin intravenously (IV) for 2 weeks	vancomycin 125 mg orally four times per day	P1: Ertapenem and ciprofloxacin P2 azithromycin	NA	12 days fidaxomicin	Amikacin, tigercylin and colistin for 14 days
	PPI	Twice daily	NA	NA	NA	NA	NA	NA
	Bowel cleansing	Once before FMT	NA	NA	NA	NA	NA	NA
Donor screening tests	See Supplementary information, Section 5	Multiple testing	screened for faecal and blood transmitted diseases	Questionnaire, blood sampling, and faecal sample analysis	NM	P1 and P2: Tested for active bacterial, viral, fungal, and parasitic infections	Screened according to a standard protocol	According to the European consensus statement
FMT	Donor Status of intervention Dilution	Unrelated Fresh stool 100 g with 100 ml sterile physiological saline	Unrelated Fresh stool 75 g processed 300 ml suspension of saline	Unrelated Cryopreserved NM	NM Frozen and encapsulated NA	Unrelated Fresh stool 100 g of faeces in 150–250 ml physiological salt	Unrelated Fresh stool 72 g of faeces in 500 ml of isotonic sodium chloride	Wife Fresh stool 30 g of faeces diluted in 150 ml of 0.9% saline
		Biliński et al. [61]	Stalenhoef et al. [62]	Grosen et al. [58]	Soto et al. [64]	Biernat et al. [60]	Bahl et al. [59]	Ueckermann et al. [64]
FMT	Dose	Single FMT	Single FMT	Single FMT and later dose of meropenem	P1:15 capsules on each of 2 successive days P2: First dose same as for P1 and the second dose was 15 capsules on each of 3 successive days	P1: three doses with 1 week gap for each P2: four doses with 1 week gap for each	Single FMT	Twice with a 2 week gap
	Route	Naso-duodenal tube	Naso-duodenal tube	Nasojejunal tube	Oral	intranasal probe	colonoscope	Nasojejuneal tube
Primary outcome		Rectal swabs collected on day 10 and 26 after FMT repeatedly did not show growth of either *K. pneumoniae* NDM + or *E. coli* ESBL+.	Negative for MDR *P. aeruginosa*, but failed to decolonize ESBL *E. coli*	*E. coli* and *K. pneumoniae* was not detected 4 and 8 month follow up testing	Both patients ultimately cleared symptomatic salmonella intestinal infection after a long course of a carbapenem and FMT.	P1: symptoms were completely resolved after the third FMT P2: antibiotic sensitive *E. coli* and *Citrobacter freundii* were detected	Resistance strains were negative at 1 week time point	Patient had no further episodes of sepsis, and blood cultures were repeatedly negative for any bacteria
Adverse effects		Loose stool and abdominal pain	Loose stool for 3 days	diarrhoea	None	None	None	None
Follow up time		1 month	3 months	12 months	P1: 4 months P2: 2 months	7 months	203 days	6 weeks

It is apparent from Table 2 that most of the AMR strains with different resistant mechanisms were susceptible to the FMT at the 1-month time point [58,59,61,63,64]. However, Stalenhoef et al. [62], reported that the patient who received single FMT failed to decolonize ESBL + *E. coli* resistant to carbapenems, gentamicin, piperacillin/tazobactam, and colistin, respectively but eradicated MDR *P. aeruginosa* successfully during the 3 months follow-up period. Similarly, Biernat et al. [60], reported that patient-1 has completely resolved the symptoms after having three consecutive doses of FMT with a 1-week interval and decolonized all the pathogens. On the contrary, the patient-2 of the same study who received four doses of FMT with a 1-week gap failed to decolonize multidrug-resistant *E. coli*, and *Citrobacter freundii.*

Though most of the studies did not mention on side effects, some patients complained of having loose stool [61,62], abdominal cramps, anorexia, and diarrhoea (Table 2) [61].

#### Case series studies

The characteristics of the case series studies (studies not discussed elsewhere) included in this review are given in Table 3 [49,50,55–57]. So far only two [49,50] randomized control trial results have been reported (Table 3). For statistical interpretation, we have divided case series studies into 2 cohorts including the case series, with and without a control arm. Additionally, we have pooled the results of all reported case series studies published so far [29–36], including the studies previously discussed elsewhere [42,43,53], to obtain a larger sample set for analysis for the FMT intervention.

**Table 3.** Characteristics of case-series studies (non-duplicate) with and without control arm included for the review.

		Bar-Yoseph et al. [55]	Davido et al. [57]	Huttner et al. [50]	Leo et al. [49]	Saidani et al. [56]
Type and place of study		Prospective cohort study, Israel	Pilot prospective monocenter study, France	Multicenter randomized superiority trial, Switzerland, Netherlands, France and Israel	Multicenter randomized clinical trial, Switzerland, Netherlands, France and Israel	A single-center case-control retrospective study
Subjects details	Age range	21.8–81.3 years	50–80 years	23–89 years	18–64 years	22–88 years
	Participants No:	15	08	22	16	10
	Male (M): Female (F)	10:5	5:3	10:12	8:8	8:2
Indication/colonization status		*K. pneumoniae*- KPC^a^ and OXA-48 *E. coli*- ESBL+^b^, NDM^c^, OXA-48, *E. cloacae*- KPC, *E. hormechai*- KPC, *K. oxytoca*- KPC, *S. marcescens*- KPC *C. freundii*- KPC	C^d^, Van-A/Van-B	*E. coli* – ESBL+, OXA and NDM, *K. pneumoniae*- ESBL+, *E. cloacae*- ESBL+, *C. freundii*- ESBL+,	ESBL-E and CPE	*K. pneumoniae*-OXA-48 and NDM-1, *E. coli* – OXA-48, *E. cloacae*-– OXA-48, *S. marcescens*- OXA-48, *A. baumanii*- OXA-24, *C. freundii*- OXA-48, *C. koseri*- OXA-48
Colonization confirmation		Cell cultures and PCR^e^ from 5 samples of rectal swab in the last 6 months	PCR method to determine Van-A/Van-B from the samples taken from a rectal swab	Stool culture		Rectal swab was taken and species identification was done through MOLDI-TOF^f^ resistance patterns via and ABSTs^g^ and RT-PCRs^h^.
Pre treatment	Antibiotic	NA^i^	NA	colistin sulphate and neomycin sulphate tablets for 5 days	Colistin/neomycin	Based upon the type of resistance strain, colistin, and aminoglycosides (amikacin or gentamicin) or sulfadiazine/fusidic acid
	PPI^j^	PPI given prior to pre-FMT fasting	PPI given 2 days prior to FMT	Omeprazole 20 mg at the evening of 1 day prior to FMT and the morning of the FMT procedure	NA	Pantoprazole 40 mg twice a day since from 2 days before to FMT
	Bowel cleansing	12 h fasting	Bowel lavage 1 day prior to FMT	NA	NA	First and second bowel wash in 5 days and 1 day before, respectively to the FMT
Donor screening tests	See Supplementary information, Section 5	Screened for infectious, metabolic and immune diseases	Done according to the French Agency for the Safety of health Products	According to the 2014 French guidance document for use of FMT in clinical trials^k^.	According to the 2014 French guidance document for use of FMT in clinical trials.	NM^l^
FMT	Donor	Unrelated	Unrelated (*n* = 2)	Unrelated	Unrelated (*n* = 7)	Unrelated
	Status of intervention sample	frozen faecal capsules	Frozen sample (−80 ^o^C)	Faecal capsules made up with 80% of glycerol	oral capsules and nasogastric tube	Aseptically prepared sample
	Dilution	NA	1–5 ml of saline per 1 g of faeces	40 g of stool in 80 ml to prepare the capsules	40 g of stool in 80 ml of saline	50 g of stool diluted in 0.9% NaCl 300–400 ml
	Dose	15 capsules/day for 2 days	Five 50 cc syringes	15 capsules in two consecutive days	15 capsules in two consecutive days in 2 centres and 80 ml of FMT preparation on a single day in remaining centres	50g/300 ml in single or double dose
	Route	Oral gavage	Nasoduodenal tube	Nasogastric administration	Nasogastric tube	Nasogastric tube or gastrostomy tube
Primary outcome at 1 month time point		CPE^m^ decolonization rat*e* > 50%	VRE decolonization rate wa*s* > 50% at one-month time point and it was 87.5% at 3 months.	40.9% decolonization rate was achieved at 1-month time point	Enterobacteriaceae was lower compared to baseline but without statistical significance	80% decolonization rate was observed at 1-month time point
Adverse effects		None	None	90% of the patients in the intervention group have at least 1 adverse event and 4 patients had severe adverse events	None	None
Follow up time		6 months	3 months	7 months	7 months	6 months

^a^*K. pneumoniae* carbapenemase; ^b^Extended spectrum β-lactamase; ^c^New-Delhi metallo-β-lactamase; ^d^Vancomycin-resistant Enterococcus; ^e^polymer chain reaction; ^f^Matrix-assisted laser desorption/ionization; ^g^antibiotic sensitivity testing; ^h^Real-time PCRs; ^i^Not applicable; ^j^proton pump inhibitor; ^k^https://ansm.sante.fr/var/ansm_site/storage/original/application/5e5e01018303790194275ded0e02353c.pdf; ^l^Not mentioned; ^m^carbapenemase-producing Enterobacteriaceae.

#### Primary outcome (decolonization of AMRs) for the case series studies without a control arm

**Figure 3.**
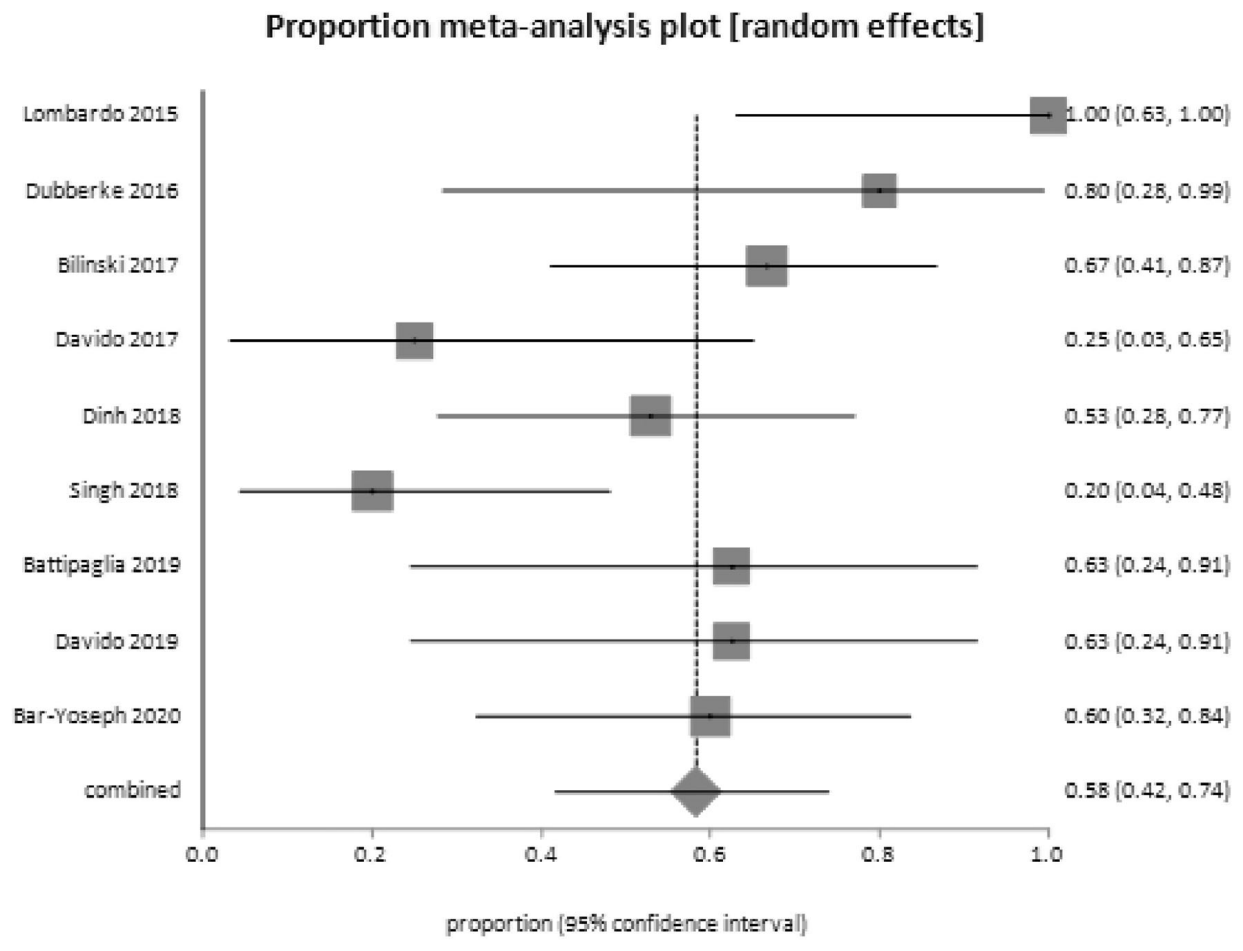
Forest plot, meta-analysis of proportions for decolonization success at 1 month.

Including the two-case series studies [55,57] listed in Table 3, nine studies were included for the meta-analysis [29,32–36]. The proportion of decolonization shows that FMT was successful in decolonizing antibiotic-resistant bacteria from the gut of participants in 55.9% (*n* = 57/102) of the cases [0.58 95% confidence interval (CI) 0.42–0.74], and presented in a forest plot (Figure 3).

#### Primary outcome (decolonization of AMRs) for the case series studies with a control arm

**Figure 4.**
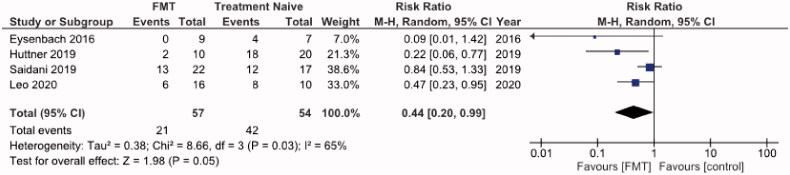
Forest plot of all case series studies reporting AMR remission at 1 month time point.

All 111 participants were included in the intention-to-treat analysis of the primary outcome, of whom 57 received FMT and 54 received no treatment. Significantly, more patients receiving donor FMT achieved clinical remission at the 1-month time point compared with those receiving control interventions, with a pooled RR of not achieving remission of 0.44 (95% CI: 0. 20–0.99) (*p* = .03) with an *I*^2^ = 65% (forest plot, Figure 4). The pooled rate of clinical remission in all 4 trials was 63.2% (*n* = 36/57) in the group receiving donor FMT and 22.2% (*n* = 12/54) in those receiving control interventions. Statistical assessment for publication bias was not performed because only 4 included trials were inadequate for funnel plots or regression-based assessments. Altogether, the forest plots obtained from the meta-analysis (Figures 3 and 4) point towards favouring FMT as an alternative treatment modality for the decolonization of AMR bacteria in the gut.

#### Assessment of heterogeneity

**Figure 5.**
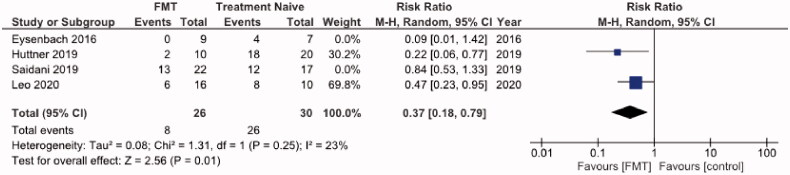
Forest plot of sub group analysis of randomized clinical trials for AMR remission at 1 month time point.

Given moderate heterogeneity (*I*^2^ = 65%), the moderate 95% CI (0.2–0.99), and the relatively small number of trials, we performed subgroup analyses for the randomized clinical trials [51,52] to explore possible explanations for the inconsistency (Figure 5). It is apparent from Figure 5 that among rigorous randomized clinical trials, FMT was associated with decolonization of AMR bacteria, with a low heterogeneity (RR 0.37; 95% CI 0.18–0.79; *I*^2^ = 23%) encouraging more randomized clinical trials in the field to ascertain a better understanding of FMT intervention.

**Figure 6.**
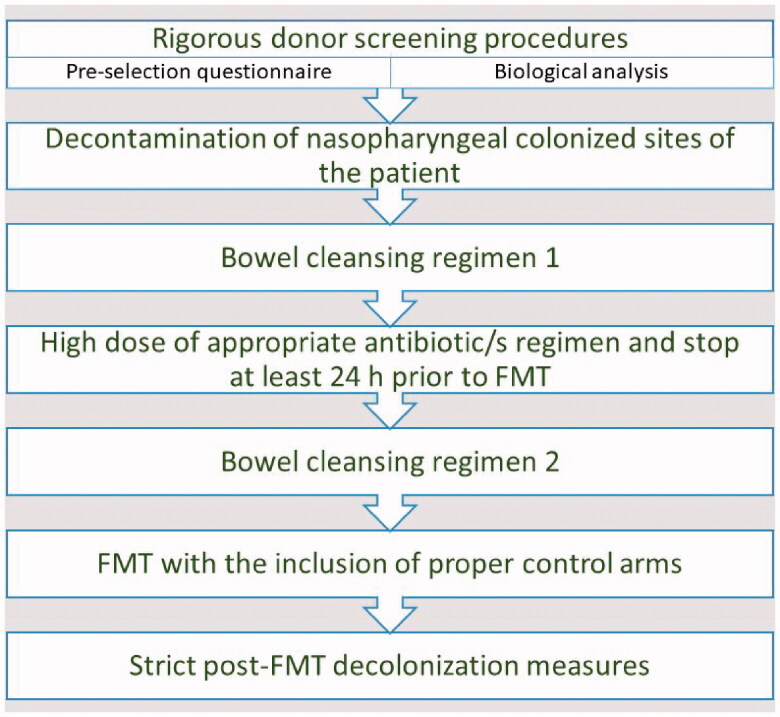
Important steps to be followed in future rigorous randomized clinical trials.

Apart from the lack of a rigorous experimental setting, other reasons also contributed to the overall moderate heterogeneity. We observed clinical heterogeneity among studies with the type of colonizing AMR bacteria (Gram-negative and Gram-positive bacteria), and their diverse resistance mechanisms including; OXA-48, OXA-24, KPC, ESBL, NDM-1, and Van A. Overall, the male patient ratio is higher than female, except in Huttner et al. [50], where there more female participants and an equal ratio was obtained in Leo et al. (Table 3) [49]. The inter-study variability of the patient preparation was also significant, such as pre-antibiotic treatment [49,50,56], use of PPIs (omeprazole or pantoprazole) before FMT [50,55–57], bowel lavage [56,57] or 12 h fasting (Table 3) [55].

Regarding the intervention of FMT, the method of sample preparation was somewhat identical. In all cases, the donors were unrelated and the intervention was in the form of capsules, except in two studies where the samples were aseptically prepared in saline and administered (Table 3) [56,57]. In most studies FMT was administered twice for each subject and on consecutive days [49,50,55,56].

#### FMT susceptible AMRs and their decolonization time points

The time frame achieved for the decolonization with respect to their resistance mechanism was also analysed as percentage decolonization at 1, 2, and 4 weeks (1 month) after FMT, and the results are presented in Table 4. The decolonization rates were within 33.3-100% at the one-month time point (Table 4) with the highest (100%) decolonization rate for AMRs in *Enterobacter hormechai* (*n* = 1), *Klebsiella oxytoca* (*n* = 1), *Citrobacter freundi* (*n* = 3), *Acinetobacter baumanii* (*n* = 1), and *Citrobacter koseri* (*n* = 1) (Table 4) and the lowest against *Serratia marcescens* with KPC (*n* = 2) (Table 4). However, it is worth noting that the sample size is inadequate to draw a robust conclusion for the effectiveness of FMT against these strains.

**Table 4.** Decolonization of different pathogens according to their resistance mechanisms at different time points.

AMRs	Resistance mechanism	Decolonization (1 W)			Decolonization (2 W)			Decolonization (1 M)			% decolonization bacteria of the bacteria at 1 M time point
		n_dec_^a^	n_tot_^b^	%_Dec_^c^	n_dec_	n_tot_	%_Dec_	n_dec_	n_tot_	%_Dec_	
*K. pneumoniae*	OXA-48	4	11	36.3	7	11	63.6	8	11	72.7	69.5
	KPC^d^	1	3	33.3	1	3	33.3	1	3	33.3	
	ESBL^e^	NM^f^	7	NA^g^	NM	7	NA	6	7	85.7	
	NDM-1^h^	1	2	50.0	1	2	50.0	1	2	50.0	
*E. coli*	NDM	NA	3	NA	1	3	33.3	1	3	33.3	52.6
	OXA/OXA-48	1	5	20.0	2	5	40.0	4	5	80.0	
	ESBL	NM	11	NA	NM	11	NA	5	11	45.5	
*E. hormechai*	KPC	NM	1	NA	1	1	100.0	1	1	100.0	100
*E. cloacae*	KPC	NM	2	NA	0	2	0.0	1	2	50.0	75
	OXA-48	1	1	100	1	1	100	1	1	100.0	
	ESBL	NM	1	NA	NM	1	NA	1	1	100.0	
*K. oxytoca*	KPC	NM	1	NA	0	1	0.0	1	1	100.0	100
*S. marcescens*	KPC	NM	2	NA	1	2	50.0	0	2	0.0	33.3
	OXA-48	0	1	0.0	NM	1	NA	1	1	100.0	
*C. freundii*	KPC	NM	1	NA	0	1	0.0	1	1	100.0	100
	OXA-48	1	1	100.0	1	1	100.0	1	1	100.0	
	NDM-1	NM	1	NA	NM	1	NA	1	1	100.0	
*A. baumanii*	OXA-24	1	1	100.0	1	1	100.0	1	1	100.0	100
*C. koseri*	OXA-48	1	1	100.0	1	1	100.0	1	1	100.0	100
VRE^i^	Van-A	NM	8	NA	NM	8	NA	5	8	62.5	62.5

^a^Number of decolonizations; ^b^total number; ^c^percentage decolonization; ^d^*Klebsiella pneumoniae* carbapenemase; eextended spectrum β-lactamase producing; ^f^not mentioned; ^g^not applicable; ^h^New Delhi metallo-β-lactamase-1; ^i^vancomycin-resistant *enterococci*.

#### Ongoing and completed clinical trials not published

We have extended our background search on different clinical trial registries and found that twenty-two (*n* = 22) more clinical trials have already been registered to investigate AMR decolonization of the gut via FMT. In that, twelve (*n* = 12), three (*n* = 3), six (*n* = 6), and one (*n* = 1) studies are randomized, non-randomized, interventional and observational clinical trials, respectively (Table 5). Out of the 12 randomized clinical trials, two studies were (*n* = 2/12) already completed, but results have not been published. A summary of all the clinical trials is given in Table 5.

**Table 5.** Characteristics of ongoing clinical trials.

Trial no./title	Primary outcome	Secondary outcome	Participants/age	Type of study (type/mask)	Country	Status
NCT02472600/Eradication of Antibiotic-resistant Bacteria Through Antibiotics and Faecal Bacteriotherapy (R-GNOSIS WP3)	Intestinal carriage of ESBL-E/CRE 35 to 48 days after randomization	adverse event, Comparison of the global microbiota composition and diversity	39/adults	Randomized/parallel open label	Switzerland	Active 02/2017 to 03/2018
NCT02922816/FMT for MDRO Colonization After Infection in Renal Transplant Recipients (PREMIX)	The safety and feasibility of using FMT in adult participants with Target MDRO colonization after infection, Change in Target MDRO Growth	Not mentioned	20/Adults	Randomized/parallel open label	USA	Active 12/2016 to 06/2021
NCT03061097/Autologous Faecal Microbiota Transplantation to Prevent Antibiotic Resistant Bacteria Colonization	Number of participants with NIH Grade ≥2 adverse events at Day 7 after randomization.	Number of patients with clearance of ARB among patients colonized at Day 28	7/Adults	Randomized/single open label	USA	Active 07/2017 to 06/2019
NCT03063437/A Trial of Encapsulated Faecal Microbiota for Vancomycin Resistant Enterococcus Decolonization	Percentage of Participants With VRE Decolonization	Percentage of Participants With VRE Infection, VRE Decolonization Among Immunocompromised Patients	9/Adults	Randomized/parallel assignment, quadraple	USA	Completed 06/2020
NCT03643887/Trial of Faecal Microbiota Transplant (FMT) for VRE and CRE Patients	Compare incidence of VRE/CRE decolonization between FMT Capsule	VRE/CRE infection at Day 3, Day 10, and Week 4 following randomization.	90/Adults	Randomized/double blind	USA	Active 09/2022 to 09/2025
NCT03802461/Effectiveness of Faecal Flora Alteration for Eradication of CPE Colonization	Incidence of CPE colonization in FMT arm vs control arm at 3 months	Incidence of CPE decolonization in FMT-treatment and non-treatment groups at 1, 6 and 12 months, Number of patients with all-cause mortality at 30 days post-randomization	40/Adults	Randomized/parallel open label	Canada	Recruiting 03/2019 to 12/2020
NCT04146337/Faecal Microbiota Transplantation for CPE	Number of participants achieving CRE eradication at 28 days	Number of participants who died by 28-day and 6-month, Number of participants with CRE bacteraemia and any bacteraemia, Adverse events	60/Adults	Randomized/parallel open label	Israel	Recruiting 10/2020 to 06/2022
NCT04181112/Faecal Transplant for MDRO Decolonization	The elimination of the target MDR organism	Compare proportions, type and timing of adverse events post-FMT, Proportions and timing of recolonization over 180 days	90/Adults	Randomized/parallel open label	Canada	Recruiting 11/2019 to 11/2023
NCT04188743/Decolonization of Gram-negative MDRO With Donor Microbiota (FMT)	Number of participants with decolonization success/failure	Side effects, Treatment effect on microbial community	150/Adults	Randomized/quadraple	Belgium	Recruiting 12/2019 to 12/2023
NCT04431934/Efficacy of a Probiotic or FMT on the Eradication of Rectal MDR Gram-negative Bacilli (MDR-GNB) Carriage (PROFTMDECOL) (PROFTMDECOL)	Proportion of patients with digestive decolonization rate defined as negative rectal swab (RS) for the target MDR-GNB (ESBL-producing *K. pneumoniae*, CPE and MDR/XDR *P. aeruginosa*) at the end of study (60 ± 7 days after the randomization)	Any changes with baseline after 1 week, 1 year	437/Adults	Randomized/parallel open label	Spain	Active/Not recruiting 06/2020 to 07/2023
NCT03061097/Autologous FMT to Prevent AMR Bacteria Colonization (RACE)	Number of Participants With Adverse Events (NIH Grade ≥2) at Day 7 After Randomization	Number of patients with clearance of ARB among patients colonized at Day 28 by PCR assay	33/Adults	Randomized/parallel multiple	Not mentioned	Completed 12/2020
ACTRN12617000561381/Gastrointestinal eradication of MDR Gram negative bacteria by FMT	Relative abundance of Gram-negative pathogenic organisms in the stool microbial community as assessed by next generation sequencing 1 year of post FMT	Number of episodes of infection, as assessed by positive culture of clinical specimen (not faeces) for resistant Gram-negative organism with same resistance mechanism	Not mentioned/Adults	Randomized/Double blind	Australia	Recruiting not mentioned
NCT02592343/A Prospective Trial of Lyophilised Faecal Microbiota Transplantation for Recurrent *C. difficile* Infection	Efficacy of lyophilised FMT for treatment of recurrent *C. difficile* infection	Evaluate treatment failure rate as defined by persistence of diarrhoea and a positive *C. difficile* toxin assay	100/Adults	Non-randomized/single open label	Canada	Completed 01/2020
NCT03050515/FMT for the treatment of recurrent urinary tract infections	Change in frequency of culture proven urinary tract infections following faecal transplant	Change in the gut microbiome following faecal transplantation measured via 16 s sequencing of stool samples	12/Adults	Non-randomized/single open label	USA	Completed 02/2020
NCT02543866/FMT as a Strategy to Eradicate Resistant Organisms	Incidence, severity, and relatedness of solicited, unsolicited, and serious adverse events	Proportion of subjects free from ESC-R intestinal colonization and recurrent ESC-R infections 2 days, 2 weeks, 4 weeks, 8 weeks, 6 months, and 12 months post-FMT	20/Adults	Non-randomized/single open label	USA	Recruiting 02/2017 to 02/2024 to
NCT02312986/FMT for MDR organism reversal	Safety of FMT in patients with recurrent MDRO infections	Proportion of subjects free from recurrent MDRO infections 30 days, 6 months, and 12 months post-FMT.	20/adults	Interventional/single open label	USA	Active 05/2015 to 06/2020
NCT02816437/FMT for MDRO Colonization in Solid Organ Transplant (FMT)	Number of participants with adverse events	Rate of MDRO decolonization, rate of recurrent MDRO infection	Not mentioned/Adults	Interventional/single open label	USA	Recruiting 07/2016 to 09/2022
NCT03029078/FMT, a Hope to eradicate colonization of patient harbouring XDR bacteria?	Negative result of the rectal swab performed, free from CRE or GRE	Study with a universal super donor in order to improve efficacy	50/Adults	Interventional/single open label	France	Active 11/2014 to 01/2024
NCT03167398/FMT for eradication of CRE	3 consecutive negative rectal samples for CRE after 1 month of trial	Not mentioned	15/Adults	Interventional/single open label	Israel	Completed 01/2020
NCT03479710/FMT for CRE/VRE	Absence of intestinal colonization of CRE/VRE	Incidence, severity and relatedness of adverse events, changes in intestinal microbiota	40/Adults	Interventional Parallel open label	Hong Kong	Recruiting 02/2018 to 12/2019
NCT03367910/FMT for MDRO UTI	FMT safety: adverse events during and after FMT	Risk of recurrent UTI post-FMT will be evaluated	60/Adults	Interventional/single open label	USA	Active 02/2018 to 06/2021
NCT04583098/The Effect of FMT on the decolonization of MDR Organisms	successful decolonization of CPE or VRE in the gut within 3 months	Not mentioned	100/Adults	Observation/Prospective cohort	Korea	Active 03/2019 to 03/2022

## Discussion

According to the contemporary guidelines from the Infectious Diseases Society of America (IDSA) and the Society of Healthcare Epidemiology of America (SHEA), as well as the European Consensus Guidelines, FMT is recommended as a second-line treatment modality against recurrent *C. difficile* infection, due to over 90% efficacy in randomized control trials [6]. Our analysis in this study points towards support of decolonization of ESKAPE pathogens with FMT intervention (Table 6). However, RCTs and sample sizes are still limited, and in addition, lack of standardized protocol and their demonstration of improvement in clinical endpoints has been inconsistent. Therefore, RCTs involving larger sample size and consensus on standardized protocols are warranted.

**Table 6.** Decolonization status of ESKAPE pathogens of the included clinical studies.

Study	ESKAPE pathogen/s	Decolonization status	
		Yes	NO
Biliński et al. [61]	*K. pneumoniae* NDM^+^ and *E. coli* ESBL	*	
Stalenhoef et al. [62]	MDR *P. aeruginosa* and MDR *E. coli*	PA^a^	** EC^b^
Grosen et al. [58]	ESBL+ *K. pneumoniae*		
Soto et al. [64]	None		
Biernat et al. [60]	*ESBL + E. coli and K. pneumoniae, E. faecium GRE*		EC^c^
Bahl et al. [59]	KPC-producing XDR *K. pneumoniae*		
Ueckermann et al. [64]	MDR *K. pneumoniae*		
Bar-Yoseph et al. [55]	*K. pneumoniae*- KPC^a^ and OXA-48 *E. coli*- ESBL+^b^, NDM^c^, OXA-48		
Davido et al. [57]	VRE		
Huttner et al. [50]	*E. coli* – ESBL+, OXA and NDM, *K. pneumoniae*- ESBL		
Leo et al. [49]	ESBL-*E.coli* and CPE		EC
Saidani et al. [56]	*K. pneumoniae*-oxa-48 and NDM-1, *E. coli* – OXA-48, *A. baumanii*- OXA-24, *E. cloacae*-– OXA-48		

^a^*P. aeruginosa*; ^b^ESBL *E.coli* was eradicated but drug sensitive *E.coli* detected upon FMT; ^c^Remission rate was not significant compared to control.

*Green colour highlighted box represents as decolonization status “yes”.

**Red colour highlighted box represents as decolonization status “No”.

The human gut contains up to 3.8 × 10^13^ bacteria that represent around 55% of stool mass [67]. Therefore, selecting a healthy donor without an intact gut microbial composition plays an important role. Similarly, the direct correlation between the effectiveness of the FMT with the pre-bowel preparation of the receptor is an equally important component for successful transplantation. Based on the studies included in this review, it is considered that an optimised FMT protocol should include: (i) rigorous donor screening procedures including, not having taken antibiotics, immunosuppressants, chemotherapy, and PPIs in recent <3 months [50,68], (ii) decontamination of nasopharyngeal colonized sites prior to FMT [56] (iii) ≥5 days prolonged treatment with high dose of appropriate antibiotic/s regimen to reduce the diversity of gut microflora and discontinue 48 h prior to prime the gut for FMT [49,50,56,58,59,61,62] (iv) two bowel cleansing regimens (one before antibiotic treatment and the other before FMT) [56] or at least (one day before FMT) [57,61] to cleanse the intestinal residues (v) adhere to strict post-FMT decolonization measures (isolation, disinfect the environment and remove catheters or other non-essential medical parts connected) and follow up (at least 3–6 months) with regular stool testing to confirm the sustainable decolonization (Figure 6).

It is worth notice that several studies [50,55–57,61] have used PPIs just before FMT, in view of protecting the transplanting microbiota from gastric acids. However, its evidence for the benefit as a pre-treatment for FMT is controversial [69], since it has been reported that PPIs are associated with an increased risk of CDI [70] and other enteric infections [71]. Also, they can alter the gut microbiota by expanding the *Enterococcus* and *Streptococcus* genera and increasing other bacterial genes associated with epithelial invasion [72,73]. Furthermore, a meta-analysis from Hong et al. [74] proved there was no statistically significant benefit from the routine use of pre-treatment with PPIs in the FMT protocol compared to no PPI-used protocols.

Even though the mechanistic studies are scanty to reveal the exact mechanism of action of FMT towards decolonization of drug-resistant bacteria, some important observations made during the studies help to speculate that FMT may have a strain-specific mechanism of action apart from general criteria like; bile-salt metabolism, GI luminal pH, and competition for resources.

Caballero et al. [66] conducted an FMT on mice co-colonized with VRE and *K. pneumoniae* those who are residing in the same region of the GI tract (lower GI tract). Unlike in most closely related species [75–78], these two strains did not show colonization resistance to each other due to nutrient competition. This may be attributed generally to their different metabolic requirements as well as their ability to switch nutrient precursors in the presence of competing strains [66]. However, investigators specifically observed that the antibiotic pre-treated control group demonstrated a thickening of the mucus layer by *K. pneumoniae* compared to VRE, indicating their high permeability towards the mucus layer, during the weak expression of host antimicrobial molecules such as RegIIIγ upon pre-antibiotic treatment [79]. This mucus infiltration, consequently increased *K. pneumoniae* translocation to mesenteric lymph nodes (mLNs) relative to VRE. Co-colonization and *K. pneumoniae* eventually increased VRE translocation too, via opening up the barrier towards mLNs. Now, the more diverse microbiota in FMT can largely colonize the intestine, while the *K. pneumoniae* and VRE are translocated towards mLNs and the complete eradication was observed in both pathogens upon FMT, which means their translocation is continuous and no replication is taking place within mLNs. Even though the exact mechanism is yet to be identified, previous studies have speculated that [80,81] cells within the colonic lamina propria, including CD103^+^ and CX3CR1^+^ dendritic cells, are believed to capture *K. pneumoniae* and carry them to mLNs.

In another *in vivo* study [65], mice were colonized to mimic two clinical conditions like the status of depleted gut microbiota and humanised mice, challenged with a high load of MDR *P. aeruginosa* (1 × 10^9^ CFU/g of faeces). In both cases, up to a 4-log_10_ reduction of MDR *P. aeruginosa* was observed upon single FMT and in a humanised model, a significant depletion of MDR carrier rate (>50%) was also observed with murine FMT at the 1-week time point. According to quantitative analysis byqRT-PCR with group-specific 16S rRNA, there was a decrease of *Enterobacteria, Enterococci*, *Bacteroides/Prevotella* species, and *Clostridia*, whereas the numbers of *Lactobacilli*, *Bifidobacteria*, and mouse intestinal *Bacteroides* were higher in the faeces derived post-FMT as compared to the pre-FMT status. Therefore, investigators hypothesised the mechanism behind the reduction of MDR *P. aeruginosa* burden was due to the higher loads of *Lactobacilli* and *Bifidobacteria* and for their pronounced production of bacteriocins and short chain fatty acids [82,83].

In a case study [59] of a 69 year-old woman referred with severe recurrent CDI and complicated by intestinal co-colonization with KPC-producing bacteria, XDR *K. pneumoniae* was successfully decolonized by a single FMT treatment. Interestingly, 16S rRNA amplicon profiling revealed *Enterobacterales* constituted 18% of the microbiota before FMT and subsequently dropped to lower levels after the FMT by increasing butyrate-producing *Faecalibacterium prausnitzii.* This species has been associated with treatment success and a mechanism for butyrate-induced reduction of intestinal inflammation and bacterial translocation of the *C. difficile* pathogen [84]. A similar observation was also made by Billinski et al. [60], where VRE decolonization was observed in a case study of an immunocompromised male patient (51 years). On the same note, the mechanism behind this decolonization was explained previously through *in vivo* mice model [85], in that the eradication may be associated with direct inhibition of VRE by a single component of healthy gut microbiota belonging to *Barnesiella* species. However, extensive studies with long-term follow-ups are warranted to determine the stability of this effect.

By using 16S rRNA metagenome sequencing, a substantial increment of *Bifidobacterium bifidum* was noted at post-FMT of a case series study conducted to decolonize CPE [55]. This species was shown to possess anti-*Enterobacteriaceae* effects [86] and anti-lysozyme activity [87], decrease biofilm formation [88], and modulate virulence gene expression [89] alone and with the combination of lactitol and *Lactobacillus acidophilus* to reduced OXA-48-producing *Enterobacteriaceae* [90]. These might explain the mechanism of action of *B. bifidum* in the decolonization of CPE in the gut.

Tavoukjian [53], have included 5 case series studies alone and according to the meta-analysis, a higher remission rate upon FMT was observed against *P. aeruginosa* while the lowest was against *K. pneumoniae* with NDM-1 and ESBL-producing strains, at the 1-month time point. These findings are in line with the analysis outcome of our systematic review, and with a comparatively lower decolonization rate for *E. coli* with NDM-1 (33.3%) and ESBL (45.5%) and *K. pneumoniae* with KPC (33.3%) (Table 4). The literature review by Amrane and Lagier [43], similarly and qualitatively, updated the recent literature.

The application of FMT for the decolonization of MDR bacteria is still in the early stage of development. Most of the eligible studies are not randomized control trials, except Huttner et al. [50] and Leo et al. [49] The effect of FMT alone is still undefined, whether the role of antibiotics and PPIs prior to FMT or other forms of priming was essential, and the possibility of spontaneous decolonization might also play a role. Therefore, further rigorous randomized control trials with the inclusion of suitable control arms are warranted to establish the therapeutic efficacy of FMT.

## Conclusion

In summary, there are limited existing studies which are generally of low quality with moderate heterogeneity, and do not allow definitive conclusions to be drawn. More rigorous RCTs with larger sample size and standardized protocols on FMTs for gut decolonization of AMR organisms are warranted.

## Abbreviations

AMR: antimicrobial resistant
AR: antibiotic resistant
CDI: *Clostridioides difficile* infection
CFU: colony forming unit
CI: confidence interval
CR: colonization resistant
EARS: European Antimicrobial Resistance Surveillance Network
ESBL: extended spectrum β-lactamase
FMT: faecal microbiota transplantation
GI: gastrointestinal
IDSA: Infectious Diseases Society of America
JBI: Joanna Briggs Institution
KPC: *Klebsiella pneumoniae* carbapenemase
MDR: multidrug-resistant
mLNs: mesenteric lymph nodes
NDM: New Delhi metallo-β- lactamase
NGS: next generation sequencing
PPIs: proton pump inhibitors
RCT: randomized clinical trials
RR: risk ratio
RT-PCR: real-time polymer chain reaction
SCFA: short chain fatty acids
SHEA: Society of Healthcare Epidemiology of America
US: United States
VRE: vancomycin-resistant enterococcus
XDR: Extensively drug-resistant
